# Systemic analysis of the DNA replication regulator origin recognition complex in lung adenocarcinomas identifies prognostic and expression significance

**DOI:** 10.1002/cam4.5238

**Published:** 2022-10-07

**Authors:** Min Tang, Juan Chen, Tian Zeng, Dong‐mei Ye, Yu‐kun Li, Juan Zou, Yu‐ping Zhang

**Affiliations:** ^1^ Department of Cardiothoracic Surgery The Second Affiliated Hospital of University of South China Hengyang Hunan People's Republic of China; ^2^ Department of Radiotherapy The Second Affiliated Hospital of University of South China Hengyang Hunan People's Republic of China; ^3^ Hunan Province Key Laboratory of Tumor Cellular & Molecular Pathology Cancer Research Institute, University of South China Hengyang Hunan People's Republic of China; ^4^ Department of Pathology The First Hospital of Nanchang City Nanchang Jiangxi People's Republic of China

**Keywords:** comprehensive bioinformatics, lung adenocarcinomas, ORC, prognostic value, public databases

## Abstract

**Background:**

DNA replication alteration is a hallmark of patients with lung adenocarcinoma (LUAD) and is frequently observed in LUAD progression. Origin recognition complex (ORC) 1, ORC2, ORC3, ORC4, ORC5, and ORC6 form a replication–initiator complex to mediate DNA replication, which plays a key role in carcinogenesis, while their roles in LUAD remain poorly understood.

**Methods:**

The mRNA and protein expression of ORCs was confirmed by the GEPIA, HPA, CPTAC, and TCGA databases. The protein–protein interaction network was analyzed by the GeneMANIA database. Functional enrichment was confirmed by the Metascape database. The effects of ORCs on immune infiltration were validated by the TIMER database. The prognostic significance of ORCs in LUAD was confirmed by the KM‐plot and GENT2 databases. DNA alteration and protein structure were determined in the cBioProtal and PDB databases. Moreover, the protein expression and prognostic value of ORCs were confirmed in our LUAD data sets by immunohistochemistry (IHC) staining.

**Results:**

ORC mRNA and protein were significantly increased in patients with LUAD compared with corresponding normal tissue samples. The results of IHC staining analysis were similar result to those of the above bioinformatics analysis. Furthermore, ORC1 and ORC6 had significant prognostic values for LUAD patients. Furthermore, the ORC cooperatively promoted LUAD development by driving DNA replication, cellular senescence, and metabolic processes.

**Conclusion:**

The ORC, especially ORC1/6, has important prognostic and expression significance for LUAD patients.

## Graphical abstract

From bioinformatics analysis aspect, systemic analysis of the expression and prognostic significance of ORCs in lung adenocarcinoma. This article was designed to help understand the roles of ORCs in the formation, development and progression of lung adenocarcinoma for further studies

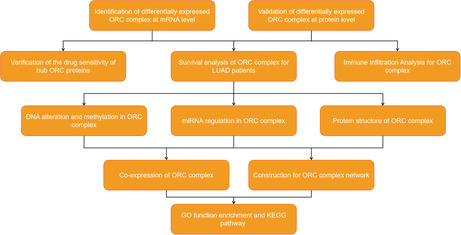

## 1 INTRODUCTION

Lung adenocarcinoma (LUAD), accounting for 60% of all lung cancers, is a common type of non‐small‐cell lung cancer (NSCLC),^1^, ^2^ which has become one of the leading causes of tumor‐related mortality worldwide.^3^ Currently, numerous studies point out both inefficient diagnosis rates and high mortality because LUAD develops silently with no specific symptoms and is difficult to treat in the advanced stage.^4^, ^5^, ^6^ Despite the development of medical science, the survival rate in advanced‐stage cancer remains 57% for patients with stage I disease and declines to 4% for those with stage IV disease.^7^ Despite regular radiographic screening, LUAD is often found in advanced stages, reducing lung cancer‐related deaths and avoiding costly treatments by improving early detection.^6^ Early stage LUAD is treated with surgery and has a favorable prognosis. In advanced LUAD, there are three main therapy options: chemotherapy, immunotherapy, and targeted therapy.^8^ Some patients with LUAD do not benefit from chemotherapy, immunotherapy, and targeted therapy due to cancerous heterogeneity. The identification of reliable biomarkers as new therapeutic targets for LUAD patients is an urgent need.

DNA replication alteration is a significant hallmark observed in multiple cancer types, such as ovarian cancer, cervical cancer, liver cancer, gastric cancer, and LUAD.^9^ Previous studies have indicated that clinical protocols targeting DNA replication‐related proteins can significantly slow lung cancer disease progression,^10^, ^11^, ^12^ revealing the great potential for the development of therapies targeting DNA replication abnormalities in patients with lung cancer. The origin recognition complex (ORC), a replication–initiator complex, binds to DNA replication origins to activate the initiation of DNA synthesis.^13^ ORC proteins can bind to potential origins to formulate the prereplication complex (pre‐RC).^14^ The assembly of the ORC, a heteromeric six‐subunit complex, is a highly orchestrated event in eucaryons. Briefly, ORC2, ORC3, and ORC5 form a complex in the cytoplasm, which can be transported to the cytoblast and bind to ORC4 and ORC6. The five‐subunit complex can further bind to ORC1 at the DNA replication origin,^15^ resulting in the initiation of DNA replication.

ORC proteins are dysregulated and accelerate the formation, development, and progression of multiple cancer types. Overexpression of ORC1 mediated by the lncRNA XIST/miR‐140‐5p axis promotes the progression of cervical cancer.^16^ In hepatocellular carcinoma, ORC1, 5, and 6 are novel biomarkers for diagnosis and prognosis.^17^ Previous studies have indicated that polo‐like kinase 1 (Plk1) phosphorylation of ORC2 mediates resistance to gemcitabine in pancreatic cancer.^18^ ORC3 can interact with the MCM complex to accelerate the progression of hepatocellular carcinoma.^19^ ORC4 gene mutation can induce B‐cell lymphoproliferative disorders.^20^ Moreover, ORC4 can also serve as a new biomarker for the diagnosis of breast cancer.^21^ In HPV‐negative head and neck squamous cell carcinoma, ORC5 is a specific biomarker for improving the diagnosis and treatment of patients.^22^ Furthermore, ORC2 or ORC5 knockdown can inhibit the recruitment of MCM2‐7 normally to chromatin in human colon cancer cell lines.^23^ ORC6 provides an excellent biomarker for gastric adenocarcinoma, colon cancer, rectal cancer, and prostate cancer.^24^, ^25^, ^26^ Inhibiting ORC6 can enhance the sensitivity of 5‐fluorouracil and cisplatin in patients with colon cancer.^27^ These results suggest the important molecular role of the ORC in the formation, development, and progression of multiple cancer types, such as hepatocellular carcinoma, gastric adenocarcinoma, colon cancer, rectal cancer, and prostate cancer. Although several studies have indicated the dysregulation of several ORCs, the significance of all ORC proteins for prognostic values and targeted treatment in patients with LUAD remains unclear.

With the establishment of public databases and the development of visual websites, the possibility of systematic analysis of ORCs in different tumor types based on bioinformatics analysis has become important in the field of cancer research. In this study, we systematically confirmed the transcriptional and posttranscriptional levels of ORCs and determined their prognostic value in LUAD. Furthermore, we also analyzed the interaction network, DNA alteration, DNA methylation, miRNA network, protein secondary and tertiary structure, immune infiltration, and functional enrichment of ORCs by bioinformatics.

## 2 METHODS

### 2.1 The mRNA and protein levels of ORCs based on public databases

The TCGA database (https://www.cancer.gov/tcga) includes data from many cancer patients, such as clinical data, genomic variation, mRNA expression, and methylation‐level expression for multiple cancer types.^28^ Furthermore, transcriptional expression analysis was performed using the UALCAN database (http://ualcan.path.uab.edu/index.html).^29^ GEPIA (http://gepia.cancer‐pku.cn/) was used in the present study to analyze the correlation among ORCs.^30^ The raw data for these analyses were included from 515 LUAD patient samples and 59 normal samples in the TCGA database LUAD data sets. The HPA (http://www.proteinatlas.org) database is an excellent tool for assessing protein levels in many cancer types and normal tissues.^31^ These analyses were conducted to understand the differential expression of ORCs in LUAD at the mRNA, protein, and methylation modification levels.

### 2.2 DNA alteration of ORCs in LUAD

The association between ORC alterations and survival outcomes in LUAD patients was confirmed by the cBioProtal database (http://www.cbioportal.org/).^32^ We included the raw data from these analyses for all 515 LUAD patients and 59 normal samples in the TCGA database LUAD data sets. This database is a open access, open source resource for the interactive exploration of multiple Cancer Genomics data sets. We used this web tool to confirm the DNA alterations of the ORC in LUAD, including the DNA alteration frequency and performed a survival analysis of LUAD patients with or without ORC alterations. Because DNA alteration is an important factor leading to abnormal gene expression, we can use this database to further clarify whether the abnormal expression of ORCs in LUAD is caused by DNA alteration.

### 2.3 Protein structure analysis for ORC proteins

The Protein Data Bank (PDB) database (https://www.rcsb.org/) is utilized in the structure of proteins for ORC complexes. We used this database to analyze the secondary and tertiary structures of proteins in the ORC complex. The structured summary was based on 7JK6 (Structure of Drosophila ORC in the active conformation).^33^ Since structure determines function, we extracted the protein structure data of ORCs using the PDB database, which further revealed the physical interaction between ORCs forming a heterohonomer to promote DNA replication.

### 2.4 Construction of the ORC complex network

GeneMANIA 3.6.0 (http://www.genemania.org) was used to construct the ORC complex network. The max resultant attributes and genes were 10 and 20, respectively. The list of genes entered into GENEMANIA was ORC1, ORC2, ORC3, ORC4, ORC5, and ORC6. Moreover, GeneMANIA assigns weights based on maximizing the connectivity between all input genes using the ‘assigned based on query gene’ strategy. The number of resultant genes, the number of resultant attributes, and the weighting method can be configured in the advanced options.^34^ The construction of a protein–protein interaction (PPI) network can further reflect the possible interaction of ORCs and explain the various biological roles of ORCs in the preparation for the subsequent gene ontology (GO) and KEGG enrichment analyses.

### 2.5 GO functional enrichment and KEGG pathway analyses

Metascape (http://metascape.org) is an excellent site for analyzing gene annotations and functional enrichment, which can be utilized to perform GO function enrichment and KEGG pathway analyses of ORC proteins and the 20 neighboring genes.^35^ GO and KEGG analyses were used to assess the relevant functional categories, with *p* and *q* values of less than 0.05 considered to be significant. GO and KEGG enrichment analyses after PPI network construction could intuitively and systematically indicate the possible molecular biological role of ORCs and its potentially mediated molecular signaling pathway, which plays a key role in predicting the molecular biological role of ORCs in the occurrence and development of LUAD.

### 2.6 Immune Infiltration Analysis of the ORC

TIMER (https://cistrome.shinyapps.io/timer/) is a simple, interactive, and effective online site for the analysis of immune infiltration in multiple cancer types.^36^ The raw data from these analyses were included from 515 LUAD patient samples in the TCGA database LUAD data sets. This site was used to further analyze the relationship between immunoinfiltration and the ORC. Immune invasion is a hot topic in current tumor treatment. Therefore, it is an important research direction to explore whether ORCs affect immune invasion. Consequently, we used the TIMER database to analyze the influence of abnormal EXPRESSION of ORCs on immune invasion in LUAD patients.

### 2.7 Survival analysis

The GENT2 database (http://gent2.appex.kr/gent2/) is a website for exploring gene expression patterns in normal and cancer samples and is utilized to perform meta‐survival analysis.^37^ The KM‐plot database (https://kmplot.com/analysis/) can be used to evaluate the effect of 54 K genes (mRNA, miRNA, and protein) on survival in multiple cancer types. The raw data for these analyses were included from 1925 LUAD patients in the TCGA database LUAD data sets. The main purpose of this tool is to conduct a meta‐analysis based on the discovery and validation of survival markers.^38^ Survival analysis is a key part of suggesting whether genes can be used as prognostic markers. We validated the prognostic value of ORCs in LUAD patients using the KM‐plot and GENT databases.

### 2.8 Clinical samples

A total of 50 LUAD and 13 normal lung tissues were surgically collected at the Second Affiliated Hospital, University of South China (Hengyang, Hunan, China) from 2015 to 2020. The collection and use of tissues were performed in keeping with the ethical standards as formulated in the Helsinki Declaration. Written informed consent was obtained from each patient, which was approved by the research ethics committee of the University of South China. The clinicopathological data are provided in Table S1.

The inclusion and exclusion criteria are outlined below: 1. Histologically proven LUAD, 2. age ≥18 years, 3. no radiotherapy or chemotherapy for LUAD, 4. written informed consent, and 5. the capacity of the patient to cooperate.

### 2.9 IHC staining

Immunohistochemistry (IHC) staining was conducted according to our previous report. IHC was performed with a two‐step detection kit (ZSBiO PV73 9000). The paraffin‐embedded tissue sections were dewaxed in xylene, rehydrated in a graded alcohol system and boiled in a high‐pressure autoclaved citric acid buffer (pH 6.0) for 15 min, and peroxidase activity was quenched with 3% hydrogen peroxide for 20 min to avoid nonspecific staining. The sections were washed three times with phosphate‐buffered saline (PBS) followed by incubation overnight with anti‐ORC1 antibody (Abcam, ab251776 at 1/1500 dilution), ORC2 antibody (Abcam, ab99277 at 1/1000 dilution), ORC3 antibody (Abcam, ab179936 at 1/200 dilution), ORC4 antibody (Abcam, ab235514 at 1/100 dilution), ORC5 antibody (ProteinTech Group, cat no. 11542‐1‐AP, at 1/400 dilution), or ORC6 antibody (Abcam, ab153993 at 1/500 dilution) at 4°C. Next, the sections were washed with PBS three times and incubated at room temperature for approximately 20 min with a reaction enhancer kit. This step was followed by three washes in PBS, incubation with secondary antibody at room temperature for 20 min, and staining with 3,3‐diaminobenzidine (DAB; Zhongshan Biotech). The sections were dehydrated and sealed after redyeing with hematoxylin.^39^

Two experienced pathologists independently assessed the percentage of positive cancer cells and their staining strength. The IHC staining intensity was scored from 0 to 2 (0, no staining; 1, weak staining; and 2, strong staining). The staining extent was scored from 0 to 4 based on the percentage of immune‐reactive cancer cells (0, 1–5, 5–25, 25–75, and >75%). A score ranging from 0 to 8 was calculated by multiplying the staining extent score by the intensity score, resulting in negative (0–4) staining or positive (5–8) staining for each example.

### 2.10 Statistical analysis

Statistical analyses were performed in the R Programming Language (version 3.6). All statistical tests were bilateral, and *p* < 0.05 was considered statistically significant.

## 3 RESULTS

### 3.1 The transcriptional and posttranscriptional levels of ORCs in LUAD

**FIGURE 1:**
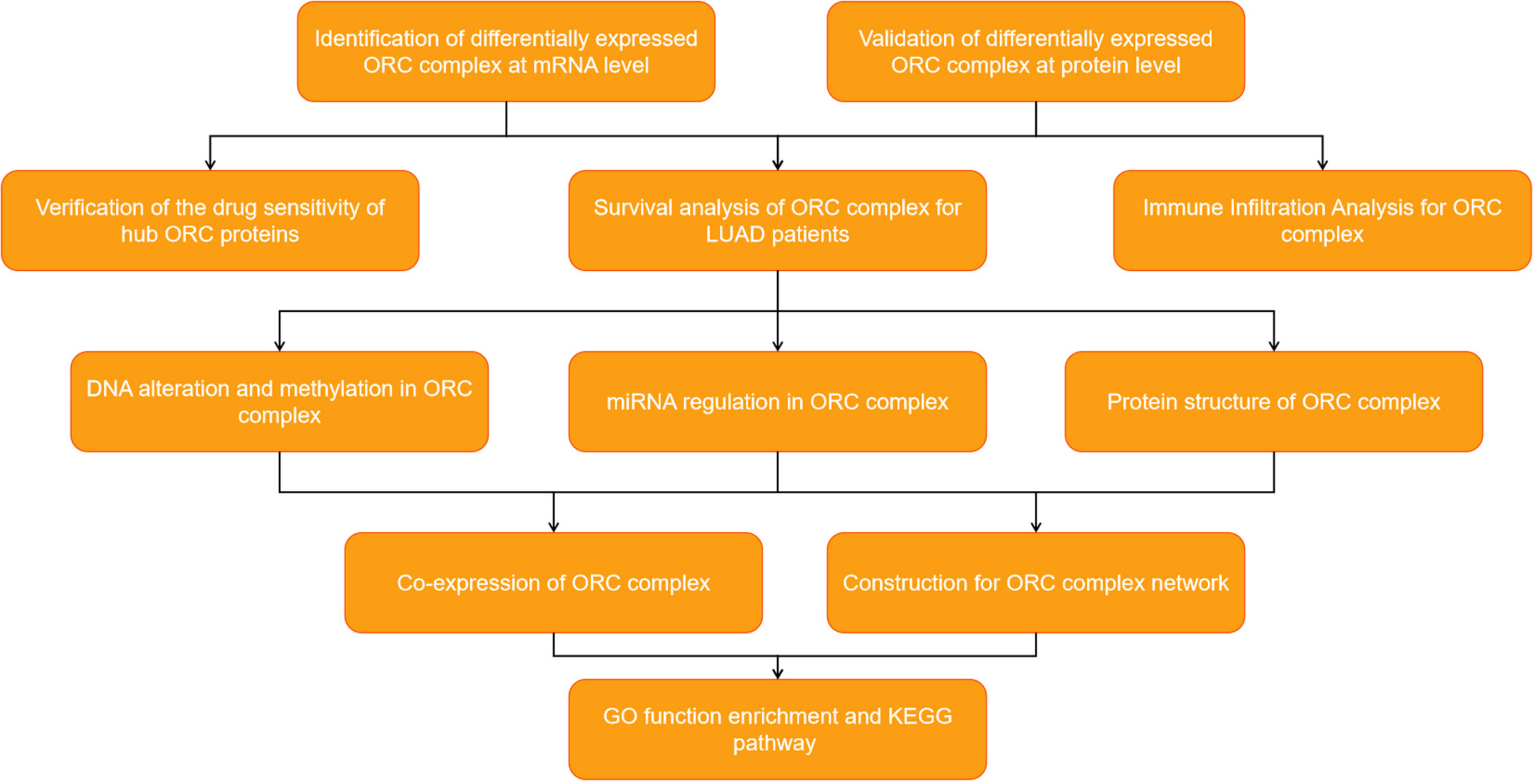
Work flow of the study.

The flow diagram of this systemic analysis is shown in Figure 1.

**FIGURE 2:**
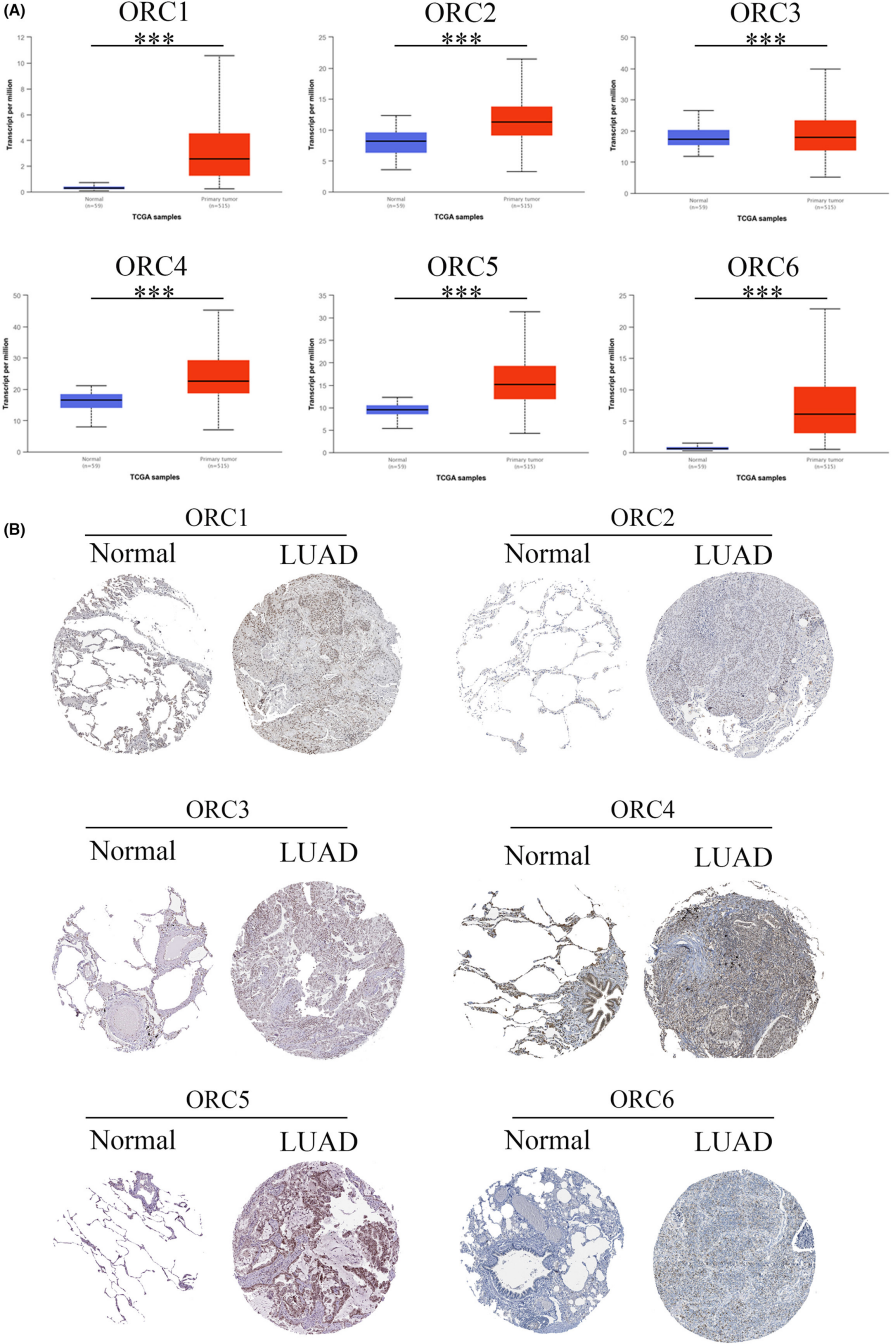
Origin recognition complex (ORC) mRNA and protein levels in lung adenocarcinoma (LUAD) based on the UALCAN and HPA databases. (A) ORC1‐6 levels in LUAD compared with normal lung samples based on the UALCAN database and (B) expression of ORC protein in LUAD compared with normal lung samples based on the HPA database. ***p < 0.001.

**FIGURE 3:**
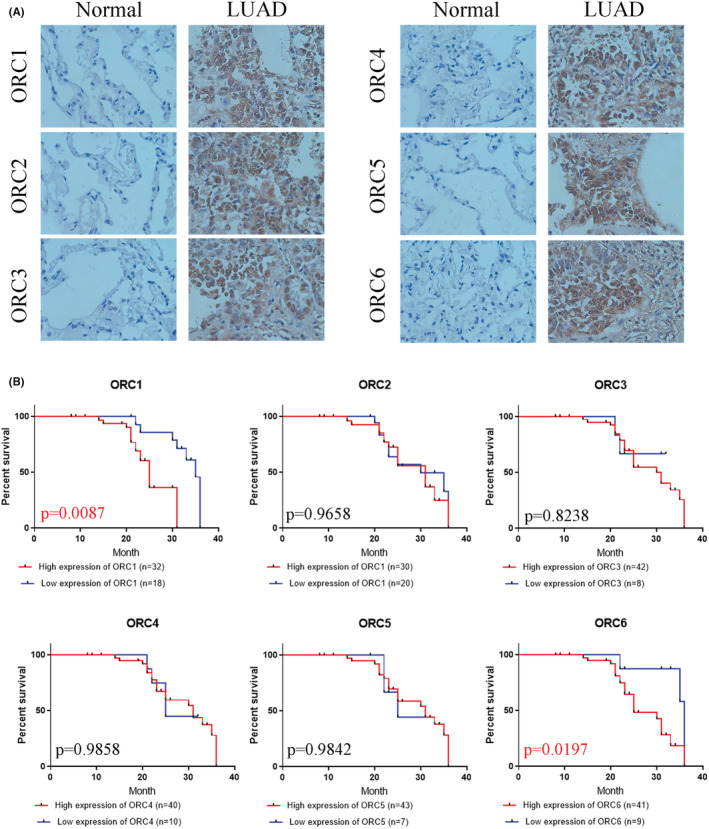
Origin recognition complex (ORC) protein expression in lung adenocarcinoma (LUAD) by immunohistochemistry staining. (A) The level of ORC in LUAD compared with normal lung samples and (B) prognostic values of the ORC levels in LUAD patients.

First, we confirmed the transcriptional level of the ORC in LUAD patients based on the UALCAN database. The results indicated that both ORC mRNA levels were significantly enhanced in LUAD patients compared with normal lung tissue samples (Figure 2A). Then, we extracted the posttranscriptional level among ORCs in LUAD patients based on the HPA database. The results showed that the IHC staining intensity of ORCs was obviously and significantly increased in LUAD tissue samples compared with normal lung tissue samples (Figure 2B). We further confirmed the expression of ORCs by IHC staining in LUAD samples and normal lung samples, which showed that ORC1‐6 expression was obviously increased in the cancer tissues of 50 LUAD patients compared with 13 normal lung samples (Figure 3A). Moreover, we also confirmed the correlation between ORC1‐6 expression and multiple clinicopathological parameters (Tables 1, 2, 3, 4, 5, 6). ORC1 and ORC4 expression was significantly correlated with tumor node metastasis (TNM) stage, lymph node metastasis, and recurrence. ORC2 was correlated with lymph node metastasis in LUAD patients. The level of ORC3 was associated with recurrence in these patients with LUAD. ORC5 expression was correlated with patient sex. The level of ORC6 was clearly associated with TNM stage and recurrence. Our results also suggested that ORC1/6 expression was negatively associated with the overall survival (OS) rate (Figure 3B). The difference between our results and the HPA results might be attributed to the difference in sample size.

**TABLE 1:** The correlation between pathological parameters and ORC1

Pathological parameters	patient number	ORC1 expression		X^2^	*p* value
		Low	High		
Diagnostic category				6.998	**0.0082**
Normal	13	10	3		
LUAD	50	18	32		
Gender				0.3438	0.5577
Male	17	6	11		
Female	33	9	24		
Age				0.6433	0.4225
<58.5	24	10	14		
≥58.5	26	8	18		
TNM stage				4.432	**0.0353**
I‐II	39	17	22		
III‐IV	11	1	10		
Smoking history				0.1019	0.7496
Yes	32	11	21		
No	18	7	11		
Lymph node metastasis				28.01	**<0.0001**
Yes	30	2	28		
No	20	16	4		
Recurrence				9.992	**0.0016**
Yes	26	4	22		
No	24	14	10		

*p* < 0.05 were shown in bold value.

Abbreviations: LUAD, lung adenocarcinomas; ORC, origin recognition complex; TNM, tumor node metastasis^1^.

**TABLE 2:** The correlation between pathological parameters and ORC2

Pathological parameters	patient number	ORC2 expression		X^2^	*p* value
		Low	High		
Diagnostic category				3.549	0.0596
Normal	13	9	4		
LUAD	50	20	30		
Gender				0.01485	0.903
Male	17	7	10		
Female	33	13	20		
Age				0.8547	0.3552
<58.5	24	8	16		
≥58.5	26	12	14		
TNM stage				1.243	0.2649
I‐II	39	14	25		
III‐IV	11	6	5		
Smoking history				1.751	0.1858
Yes	32	15	17		
No	18	5	13		
Lymph node metastasis				8.681	**0.0032**
Yes	30	7	23		
No	20	13	7		
Recurrence				0.8547	0.3552
Yes	26	12	14		
No	24	8	16		

*p* < 0.05 were shown in bold value.

Abbreviations: LUAD, lung adenocarcinomas; ORC, origin recognition complex; TNM, tumor node metastasis.

**TABLE 3:** The correlation between pathological parameters and ORC3

Pathological parameters	patient number	ORC3 expression		X^2^	*p* value
		Low	High		
Diagnostic category				18.76	**<0.0001**
Normal	13	10	3		
LUAD	50	8	42		
Gender				0.05199	0.8196
Male	17	3	14		
Female	33	5	28		
Age				0.01526	0.9017
<58.5	24	4	20		
≥58.5	26	4	22		
TNM stage				1.333	0.2482
I‐II	39	5	34		
III‐IV	11	3	8		
Smoking history				0.8102	0.3681
Yes	32	4	28		
No	18	4	14		
Lymph node metastasis				0.3968	0.5287
Yes	30	4	26		
No	20	4	16		
Recurrence				4.809	**0.0283**
Yes	26	7	19		
No	24	1	23		

*p* < 0.05 were shown in bold value.

Abbreviations: LUAD, lung adenocarcinomas; ORC, origin recognition complex; TNM, tumor node metastasis.

**TABLE 4:** The correlation between pathological parameters and ORC4

Pathological parameters	patient number	ORC4 expression		X^2^	*p* value
		Low	High		
Diagnostic category				11.87	**0.0006**
Normal	13	9	4		
LUAD	50	10	40		
Gender				0.08913	0.7653
Male	17	3	14		
Female	33	7	26		
Age				0.02003	0.8874
<58.5	24	5	19		
≥58.5	26	5	21		
TNM stage				24.5	**<0.0001**
I‐II	39	2	37		
III‐IV	11	8	3		
Smoking history				1.063	0.3024
Yes	32	5	27		
No	18	5	13		
Lymph node metastasis				4.688	**0.0304**
Yes	30	3	27		
No	20	7	13		
Recurrence				3.926	**0.0475**
Yes	26	8	18		
No	24	2	22		

*p* < 0.05 were shown in bold value.

Abbreviations: LUAD, lung adenocarcinomas; ORC, origin recognition complex; TNM, tumor node metastasis.

**TABLE 5:** The correlation between pathological parameters and ORC5

Pathological parameters	patient number	ORC5 expression		X^2^	*p* value
		Low	High		
Diagnostic category				30.04	**<0.0001**
Normal	13	12	1		
LUAD	50	7	43		
Gender				5.081	**0.0242**
Male	17	5	12		
Female	33	2	31		
Age				0.2726	0.6016
<58.5	24	4	20		
≥58.5	26	3	23		
TNM stage				0.2823	0.5952
I‐II	39	6	33		
III‐IV	11	1	10		
Smoking history				1.666	0.1968
Yes	32	6	26		
No	18	1	17		
Lymph node metastasis				3.35	0.0672
Yes	30	2	28		
No	20	5	15		
Recurrence				0.2726	0.6016
Yes	26	3	23		
No	24	4	20		

*p* < 0.05 were shown in bold value.

Abbreviations: LUAD, lung adenocarcinomas; ORC, origin recognition complex; TNM, tumor node metastasis.

**TABLE 6:** The correlation between pathological parameters and ORC6

Pathological parameters	patient number	ORC6 expression		X^2^	*p* value
		Low	High		
Diagnostic category				21.13	**<0.0001**
Normal	13	11	2		
LUAD	50	9	41		
Gender				0.6785	0.4101
Male	17	2	15		
Female	33	7	26		
Age				0.05559	0.8136
<58.5	24	4	20		
≥58.5	26	5	21		
TNM stage				9.35	**0.0022**
I‐II	27	9	18		
III‐IV	23	0	23		
Smoking history				1.822	0.1771
Yes	32	4	28		
No	18	5	13		
Lymph node metastasis				1.107	0.2928
Yes	30	4	26		
No	20	5	15		
Recurrence				7.352	**0.0067**
Yes	26	1	25		
No	24	8	16		

*p* < 0.05 were shown in bold value.

Abbreviations: LUAD, lung adenocarcinomas; ORC, origin recognition complex; TNM, tumor node metastasis.

### 3.2 Prognostic values of the ORC Complex in LUAD patients

**FIGURE 4:**
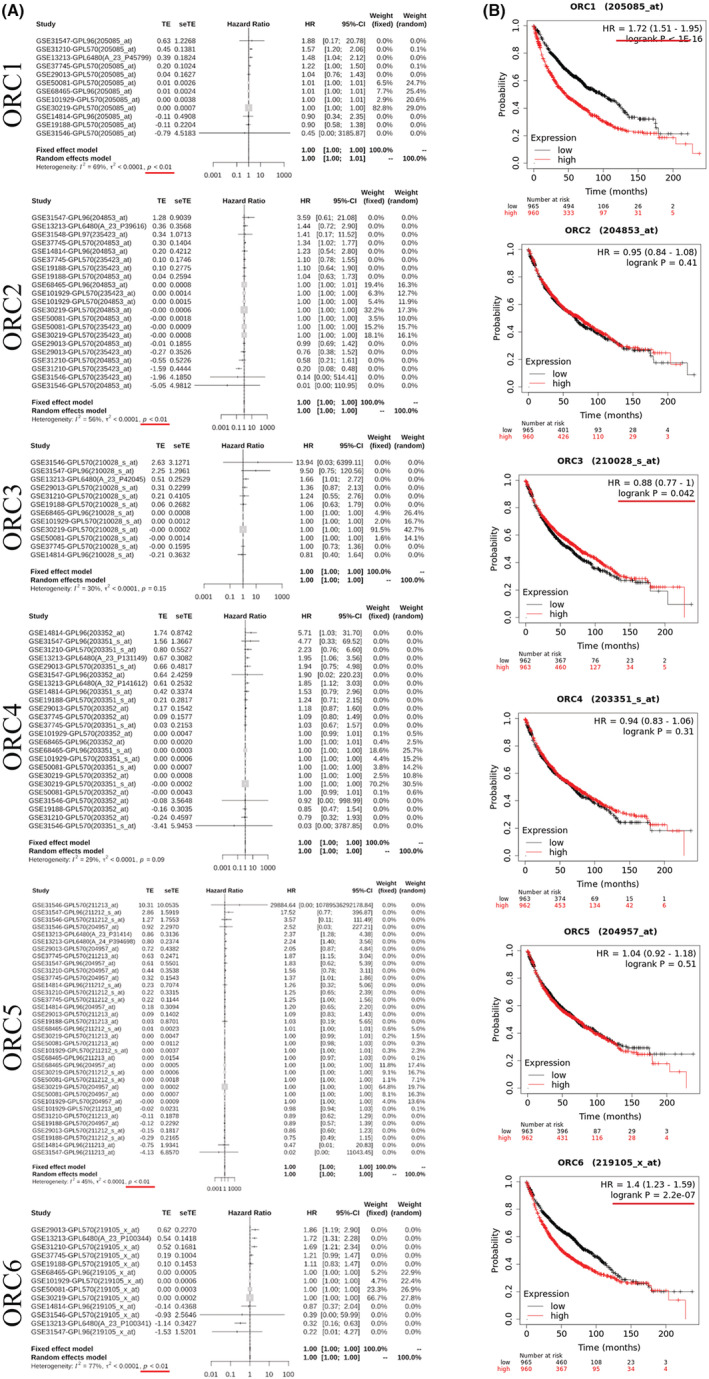
GENT2 meta‐survival analysis and Kaplan–Meier Plotter survival analysis. (A) Meta‐survival analysis for origin recognition complexes (ORCs) based on the GENT2 database and (B) survival analysis of ORCs based on the Kaplan–Meier plotter database.

From the above analysis, we found that the prognostic values of these ORCs were obviously different based on the transcriptional and posttranscriptional levels. To further validate the prognostic value of these ORCs, we conducted survival analyses of the ORCs used by the KM plot and GEO databases. The meta‐survival analyses showed that ORC1, ORC2, ORC5, and ORC6 had prognostic values (Figure 4A). KM‐plot database analysis showed that the overall survival probabilities of ORC1, ORC3, and ORC6 were significant (Figure 4B). Moreover, we used Cox analysis to determine which ORC was the major influencing factor of overall survival in LUAD patients. The results showed that ORC1/2/6 played a significant role in the development and progression of LUAD (Figure S1). Taken together, the prognostic values of ORC1 and ORC6 were more significant than those of other ORCs in LUAD patients.

### 3.3 The underlying mechanism of ORC complex regulation based on bioinformatics analysis

**FIGURE 5:**
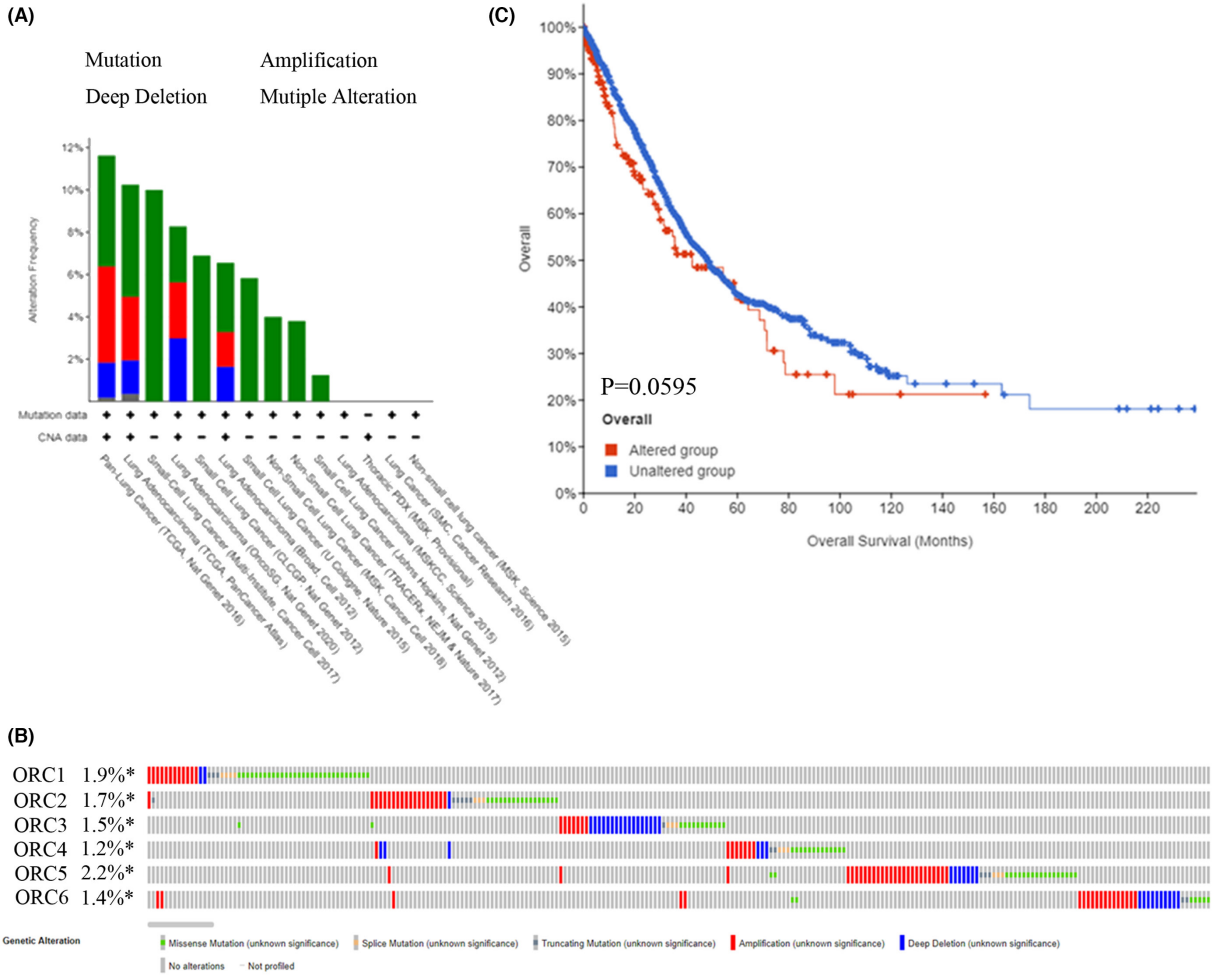
Alteration of origin recognition complexes (ORCs). (A) DNA alteration of ORCs in lung adenocarcinoma (LUAD), (B) frequency of the ORC in LUAD based on the cBioProtal database, and (C) overall survival in LUAD patients with or without ORC alterations.

**FIGURE 6:**
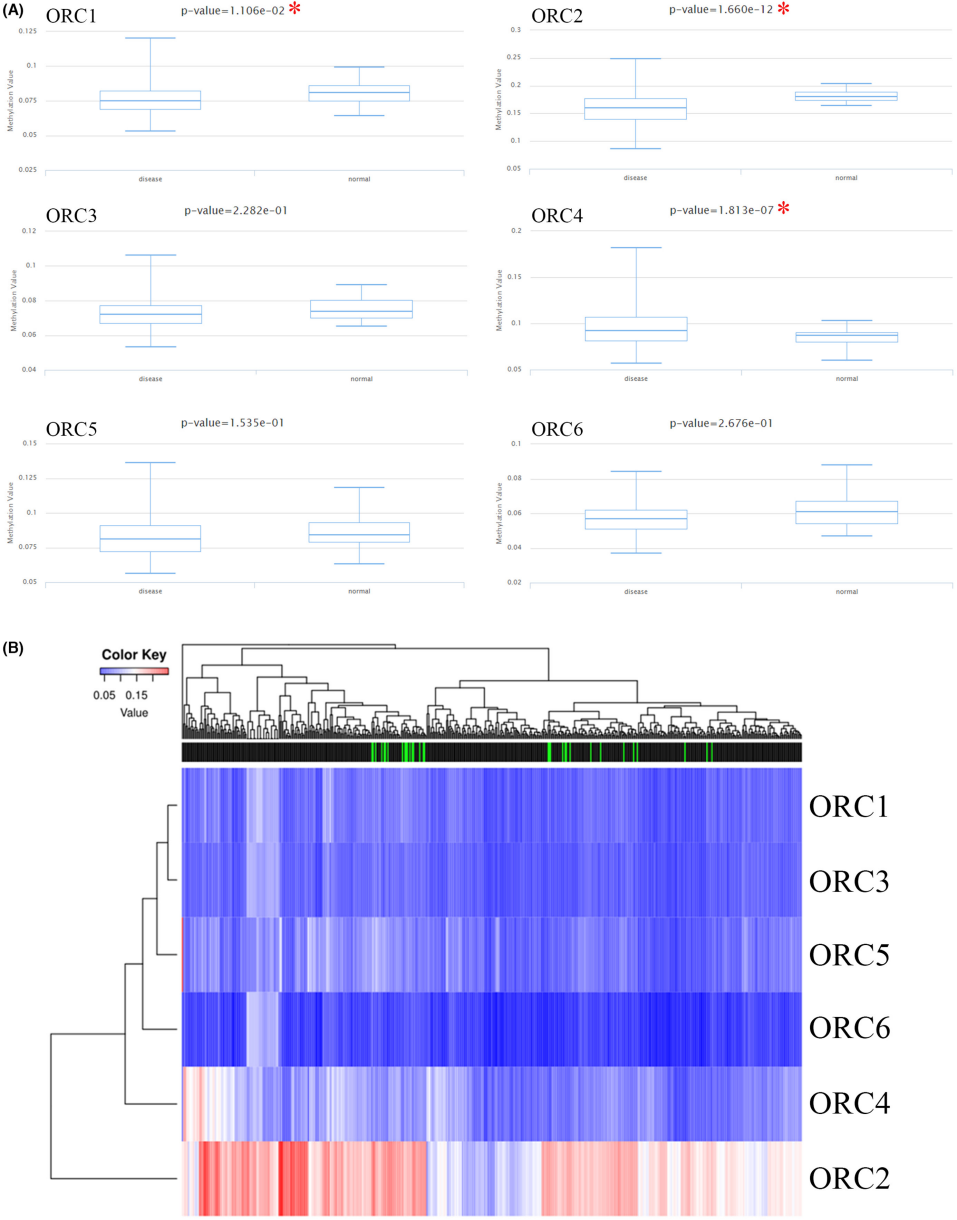
DNA methylation and miRNA network of origin recognition complexes (ORCs). (A) DNA methylation of ORCs in lung adenocarcinoma (LUAD) compared with normal lung samples based on the TCGA database and (B) heatmap of ORC methylation in LUAD and normal lung samples.

At the DNA level, we explored the alteration level in the ORC complex based on the cBioProtal database. The lung cancer dataset showed that the DNA alteration percentages of the ORC complex were 1.9% (ORC1), 1.7% (ORC2), 1.5% (ORC3), 1.2% (ORC4), 2.2% (ORC5), and 1.4% (ORC6) (Figure 5A,B). Then, we further confirmed the survival rate between the ORC complex alteration group and the no alteration group (Figure 5C), which indicated that the DNA alteration was not correlated with the prognosis outcome in LUAD patients. Moreover, we also investigated the DNA methylation level of ORCs in LUAD based on the TCGA database. The results indicated that the methylation level was significantly decreased in the CpG promoter of ORC1 and ORC2 but significantly increased in the CpG promoter of ORC4 in LUAD tissue samples compared with normal lung tissue samples (Table 7; Figure 6A). However, the heatmap with hierarchical clustering of CpG methylation among ORCs was not significant (Figure 6B), which indicated that the methylation profiles could not be used to distinguish normal samples from cancers. These results indicated that these ORCs were regulated by DNA methylation rather than alteration at the DNA level.

**TABLE 7:** The methylation of ORCs in LUAD

Gene	Disease name	Genomic region	Transcript	*p* value	*p* value < 0.05?	FDR
ORC1	LUAD	chr1:52869643‐52872143	NM_001190819	1.11E‐02	Yes	−0.004
ORC2	LUAD	chr2:201827924‐201830424	NM_006190	1.66E‐12	Yes	−0.021
ORC3	LUAD	chr6:88297784‐88300284	NM_012381	2.28E‐01	No	−0.002
ORC4	LUAD	chr2:148777816‐148780316	NM_001190879	1.81E‐07	Yes	0.012
ORC5	LUAD	chr7:103847995‐103850495	NM_002553	1.54E‐01	No	−0.004
ORC6	LUAD	chr16:46721557‐46724057	NM_014321	2.68E‐01	No	−0.002

Abbreviations: FDR, false discovery rate; LUAD, lung adenocarcinomas; ORC, origin recognition complex; TNM, tumor node metastasis.

### 3.4 Functional enrichment and pathway analyses for the ORC in LUAD

**FIGURE 7:**
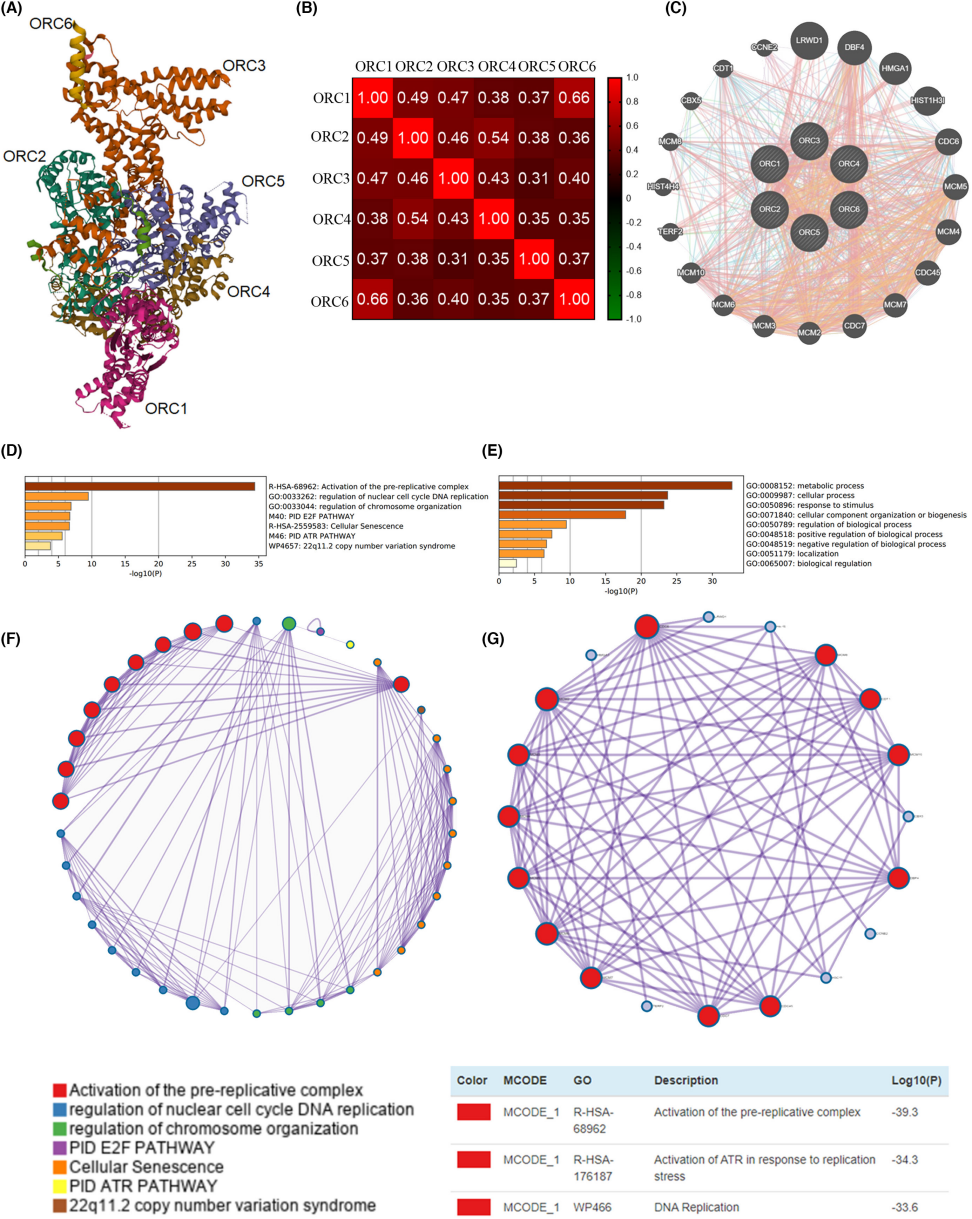
Coexpression, interaction, function enrichment, and KEGG pathway enrichment. (A) Tertiary structure of origin recognition complexes (ORCs), (B) Spearman's correlation analysis of ORCs, (C) protein–protein interaction network among ORCs, (D) pathway and process enrichment analysis, (E) the top‐level gene ontology biological processes, (F) networks for pathway and process enrichment analysis, and (G) networks for protein–protein interaction enrichment analysis.

Subsequently, the structural models of six ORC subunits were also constructed by the PDB database (Figure 7A), which indicated that these ORCs were able to bind to each other. We also mined the mRNA data of ORCs in LUAD patients to analyze the correlations among these ORC complexes based on the TCGA database (Figure 7B). This result indicated a positive and significant correlation between ORC1/2/3/4/5/6 and other ORCs, especially ORC1–ORC6 (*R* = 0.66) and ORC2–ORC4 (*R* = 0.54). Next, the PPI networks of the ORC were constructed by GeneMANIA, including ORC1, ORC2, ORC3, ORC4, ORC5, ORC6, LRWD1, DBF4, HMGA1, HIST1H3I, CDC6, MCM5, MCM4, CDC45, MCM7, CDC7, MCM2, MCM3, MCM6, MCM10, TERF2, HIST4H4, MCM8, CBX5, CDT1, and CCNE2 (Figure 7C). Then, we used these genes generated by GeneMANIA tools to further analyze the GO functional enrichment and KEGG pathway analyses by the Metascape database. Pathway and process enrichment analysis indicated that these genes had an important effect on the activation of the prereplicative complex, regulation of nuclear cell cycle DNA replication, regulation of chromosome organization, PID E2F pathway, cellular senescence, PID ATR pathway, and 22q11.2 copy number variation syndrome (Figure 7D). The top‐level GO biological processes showed that these genes were enriched in multiple biological processes, such as the metabolic process, cellular process, response to stimulus, cell component organization or biogenesis, regulation of the biological process, positive regulation of the biological process, negative regulation of the biological process, localization, and biological regulation (Figure 7E). Then, we also constructed networks for pathway and process enrichment analysis (Figure 7F) and PPI enrichment analysis (Figure 7G), which showed the interaction among these GO enrichments and KEGG pathway analyses.

### 3.5 The correlation between ORC complex expression and immune infiltration

**FIGURE 8:**
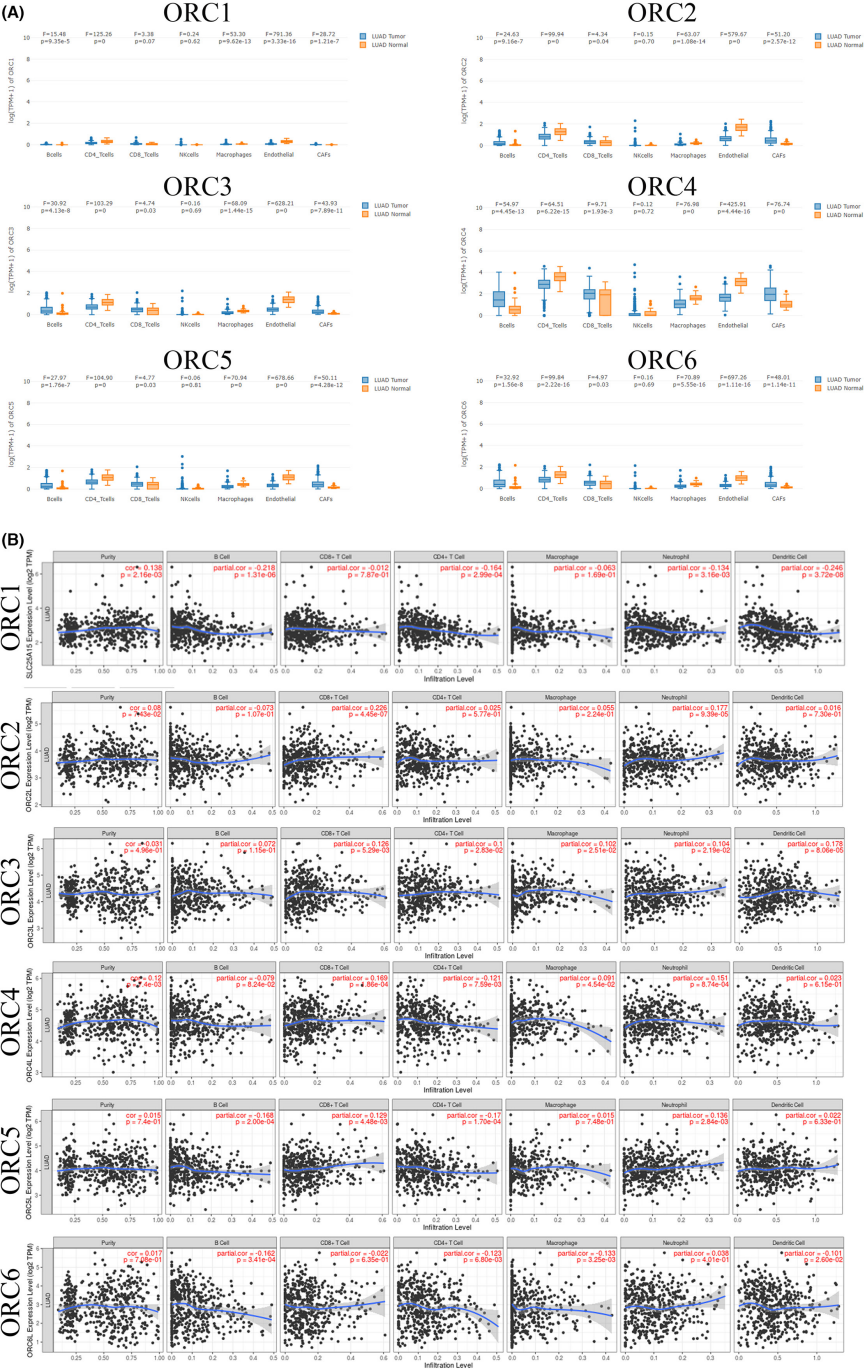
Immune infiltration of the origin recognition complex (ORC) in lung adenocarcinoma. (A) ORC expression in each immune cell type and cell type‐level differential expression analysis results and (B) cancer purity and immune infiltration.

Due to the significant role of tumor‐infiltrating immunity in carcinogenesis and its impact on prognosis, we also confirmed the immune infiltration of the ORC in LUAD based on the GEPIA database. Our results indicated that the expression of ORC1, ORC2, ORC3, ORC4, ORC5, and ORC6 was different in the seven common types of tumor‐infiltrating immune cells, such as B cells, CD4 T cells, CD8 T cells, NK cells, macrophages, endothelial cells, and cancer‐associated fibroblasts (Figure 8A). Furthermore, we found that ORC1 was correlated with purity (*p* = 2.16e‐03, cor = 0.138), B cells (*p* = 1.31e‐06, cor = −0.218), CD4^+^ T cells (*p* = 2.99e‐04, cor = −0.164), neutrophils (*p* = 3.16e‐03, cor = −0.134), and dendritic cells (*p* = 3.72e‐08, cor = −0.246). ORC2 expression was associated with CD8^+^ T cells (*p* = 4.45e‐07, cor = 0.226) and neutrophils (*p* = 9.39e‐05, cor = 0.177). The expression of ORC3 was correlated with CD8^+^ T cells (*p* = 5.29e‐03, cor = 0.126), CD4^+^ T cells (*p* = 2.83e‐02, cor = −0.1), macrophages (*p* = 2.51e‐02, cor = 0.102), neutrophils (*p* = 2.19e‐02, cor = 0.104), and dendritic cells (*p* = 8.06e‐05, cor = 0.178). The ORC4 mRNA level was correlated with purity (*p* = 7.4e‐03, cor = 0.12), CD8^+^ T cells (*p* = 1.86e‐04, cor = 0.169), CD4^+^ T cells (*p* = 7.59e‐03, cor = −0.121), macrophages (*p* = 4.54e‐02, cor = 0.091), and neutrophils (*p* = 8.74e‐04, cor = 0.151). ORC5 was correlated with B cells (*p* = 2e‐04, cor = −0.168), CD8^+^ T cells (*p* = 4.48e‐03, cor = 0.129), CD4^+^ T cells (*p* = 1.70e‐04, cor = −0.17), and neutrophils (*p* = 2.84e‐03, cor = 0.136). ORC6 was also correlated with B cells (*p* = 3.41e‐04, cor = −0.162), CD4^+^ T cells (*p* = 6.80e‐03, cor = −0.123), macrophages (*p* = 3.25e‐03, cor = −0.133), and dendritic cells (*p* = 2.60e‐02, cor = −0.101) (Figure 8B). Therefore, these ORCs were closely associated with immune infiltration to varying degrees in LUAD patients.

### 3.6 Verification of the drug sensitivity of hub ORC proteins

Finally, we analyzed the drug sensitivity of hub ORC proteins. Our results indicated that ORC1 (SLC25A15) and ORC2 (SLC25A2) were closely associated with chemotherapy resistance based on the GSCALite database (Figures S2 and S3). Hence, these results indicated that ORC1 and ORC2 could be potential therapeutic targets for LUAD patients.

## 4 DISCUSSION

It has already been reported that the ORC can promote the initiation of DNA synthesis,^13^, ^40^ but there are data in the literature on its interaction with cancer development and progression. In previous studies, the heterohexameric complex composed of ORC1‐6 has been systematically analyzed in hepatocellular carcinoma.^13^ Dysregulation of ORC proteins has been found in multiple cancer types, including cervical cancer,^16^ colon cancer,^23^ leukemia,^41^ glioma,^42^ tongue cancer,^43^ head and neck squamous cell carcinoma,^22^ liver cancer,^17^ and gastric cancer.^24^ Nevertheless, little is known about the prognostic and expression significance of ORC1‐6 in LUAD.

In this study, we found that ORC proteins were significantly increased in LUAD tissue samples compared with normal lung tissue samples at the transcriptional and posttranscriptional levels. Yitong Zhang et al. indicated that ORC1 levels increased according to the pathological stages of LUAD and could be utilized as a therapeutic target in LUAD treatment by inhibiting stemness features.^44^ Susana Gonzalez and colleagues indicated that repression of the INK4/ARF locus could suppress the oncogenic activity of Cdc6 in lung cancer, which could inhibit the formation of multiprotein complexes, including ORC2, Cdc6, and MCMs.^45^ We also found that the expression levels of ORC might be regulated by CpG methylation at the DNA level, miRNA regulation at the mRNA level, and chemical modification at the protein level. Chen et al. found that the lncRNA XIST/miR‐140‐5p/ORC1 axis markedly decreased cervical cancer cell proliferation, blocked the cell cycle, and suppressed cell metastasis.^16^ Kohzaki et al. found that ORC1/2 was regulated by H3K4 methylation in Drosophila melanogaster.^46^ These studies indicated that ORC1 and ORC2 might be potential oncogenes in the development and progression of lung cancer. Regrettably, how ORC3/4/5/6 and LUAD cells perform crosstalk using molecular‐based language has been largely overlooked.

A previous study suggested that the ORC could connect to DNA replication sites in the late G1 phase and early S phase in mammalian cells.^47^ The ORC, as a replication–initiator complex, can bind to Cdc6 and Cdt1, which can ultimately combine with the loading of two MCM complexes.^48^ The MCM2‐7 double hexamer promotes DNA replication at the replication origin.^49^ Moreover, the molecular DNA synthesis by the ORC‐Cdc6‐Cdt1‐MCM complex is highly conserved, especially in mammalian species and cell types.^40^ ORC1 was found to control centriole and centrosome reduplication to promote DNA replication in osteosarcoma progression.^50^ Wang et al. confirmed that the sumoylation of ORC2 was important for the smooth transition into mitosis.^51^ ORC2 also regulates telomere homeostasis^52^, ^53^ and chromosome condensation^54^ in eukaryotes, which indicates that the function and modification of ORC proteins are crucial for DNA replication. ORC3 can induce the organization of higher chromatin structures by interacting with HP1 at heterochromatin foci.^55^ ORC4/6 can interact with ENY2 to bind to the C2H2 zinc fingers of insulator protein Su (Hw), which opens chromatin regions and accelerates the recruitment of the ORC to chromatin.^56^, ^57^, ^58^ ORC5 could interact with GCN5/KAT2A, a significant histone acetyltransferase, inducing origins of replication that are more accessible for activation.^59^ Moreover, the multimmonoubiquitylation of ORC5 could accelerate the opening of the local origin chromatin environment to promote DNA replication.^60^ Furthermore, Bernhard Suter found that ORC‐dependent DNA replication was regulated by the histone acetyltransferases Hat1p/Hat2p in yeast.^61^ In conjunction with our protein tertiary structure, ORC proteins can bind to each other to form a hexamer. The levels of the six ORC proteins were both enhanced significantly and highly correlated with each other in LUAD. Taken together, these results indicated that the ORC might promote DNA replication in LUAD progression.

In our functional enrichment and pathway analyses, in addition to the regulation of nuclear cell cycle DNA replication, which has been reported by many studies, ORC proteins also showed other important molecular biological effects, such as cellular senescence and metabolic processes. Recent literature has suggested that ORC1 can activate the ERK and JNK signaling pathways to enhance proliferation and metastasis in glioma.^42^ However, Saha and colleagues found that transient expression of ORC1 rapidly induced p53‐independent apoptosis, and ORC1 accumulated perinuclearly rather than uniformly throughout the nucleus.^62^ ORC2 can properly induce chromatin segregation at the G2/M phase by interacting with the centrosome and centromere.^52^ SUMOylated ORC2 can recruit a histone demethylase to convert H3K4me3 to H3K4me2, preventing the rereplication of heterochromatin DNA.^51^ Kato et al. found that ORC5 maintained the stability of the genome throughout the cell cycle in *Schizosaccharomyces pombe*.^63^ ORC6 has been shown to enhance drug resistance in patients with colon cancer, especially resistance to 5‐fluorouracil and cisplatin.^27^ Moreover, ORC6 promotes proliferation, migration, and invasion of hepatocellular carcinoma cells.^64^ Junsuo Kan et al. also found that the ORC can collaborate with SPP1 to upregulate H3K4me3 in *Saccharomyces cerevisiae*. Because the ORC is functionally highly conserved between yeast and humans, its effects on histone methylation are highly similar.^65^ In our IHC staining results, we found that ORC1/3/4/6 was significantly correlated with recurrence, and ORC1/2/4 was obviously associated with lymph node metastasis in patients with LUAD. In summary, these results suggest that ORC proteins not only participate in DNA replication but also have other molecular functions.

It is interesting to note that the enhanced dependence of the ORC‐mutant cells on CDC6 for viability increases the likelihood that CDC6, perhaps with ORC6, can under exceptional circumstances carry out the function of the ORC ring in recruitment and loading of the MCM2–7 prehelicase complex around DNA.^23^ ORC5 is frequently deleted in acute myeloid leukemia (AML) and myelodysplastic syndrome (MDS) and appears to be a candidate tumor suppressor gene for these diseases. However, ORC5 does not function as a tumor suppressor in these diseases, thus implying that DNA alteration has little effect on ORC5.^66^ We also confirmed the DNA alteration in ORC proteins. The DNA alterations of ORC1/2/3/4/5/6 were 1.9%, 1.7%, 1.5%, 1.2%, 2.2%, and 1.4%, respectively. However, ORC alterations were not significantly correlated with the prognosis of patients with LUAD.

There are several limitations to this study. First, our study was based on multiple public databases, thus requiring further verification of the expression of these ORCs in LUAD cell lines and human LUAD samples. Then, the potential molecular mechanisms (apart from DNA replication), especially in cellular senescence and metabolic processes, must be further clarified in additional experiments. These results will help to elucidate the role of ORCs and relevant signaling pathways in the formation, development, and progression of LUAD.

## 5 CONCLUSION

In the present study, we systematically and comprehensively examined the transcriptional and posttranscriptional ORCs and their prognostic significance in LUAD. We also confirmed the regulation of ORCs at the DNA level (DNA alteration and methylation), mRNA level (miRNA regulation), and protein level (chemical modification). Furthermore, we analyzed the coexpression, interaction network, structural models, enrichment pathways, immune infiltration, and drug sensitivity of ORC proteins. The results showed that the transcriptional and posttranscriptional ORCs were significantly increased in LUAD samples compared with normal samples. The mRNA and protein levels of ORC1 and ORC6 were significantly correlated with LUAD prognosis. Moreover, apart from DNA replication, the ORC may have other important biological functions, such as immune infiltration, cellular senescence, and metabolic processes. In summary, ORCs could serve as new biomarkers with prognostic and expression significance in patients with LUAD, which will facilitate the development of more appropriate clinical treatment in the future.

## AUTHOR CONTRIBUTIONS

M Tang, J Zou, and T Zeng analyzed the data. DM Ye, J Chen, and T Zeng used online tools. J Zou and YP Zhang designed the project, selected the analyzed results, and wrote the paper. All the authors contributed to the article and approved the submitted version.

## FUNDING INFORMATION

The present study was supported by the Hunan Provincial Health Commission 2019 Annual Scientific Research (grant no. C2019098).

## CONFLICT OF INTEREST

The authors declare that the research was conducted in the absence of any commercial or financial relationships that could be construed as a potential conflict of interest.

## ETHICS APPROVAL AND CONSENT TO PARTICIPATE

Not applicable.

## CONSENT FOR PUBLICATION

Not applicable.

## Supporting information


Figure S1

Figure S2

Figure S3

Table S1

## Data Availability

The data sets presented in this study can be found in online repositories. The names of the repository/repositories and accession number(s) can be found in the article/supplementary material.
