# Supplementary figures and images for: Systemic analysis of the DNA replication regulator origin recognition complex in lung adenocarcinomas identifies prognostic and expression significance
Source: Cancer Med. 2022 Oct 7;12(4):5035–54. doi: 10.1002/cam4.5238 (PMC9972100; doi:10.1002/cam4.5238)

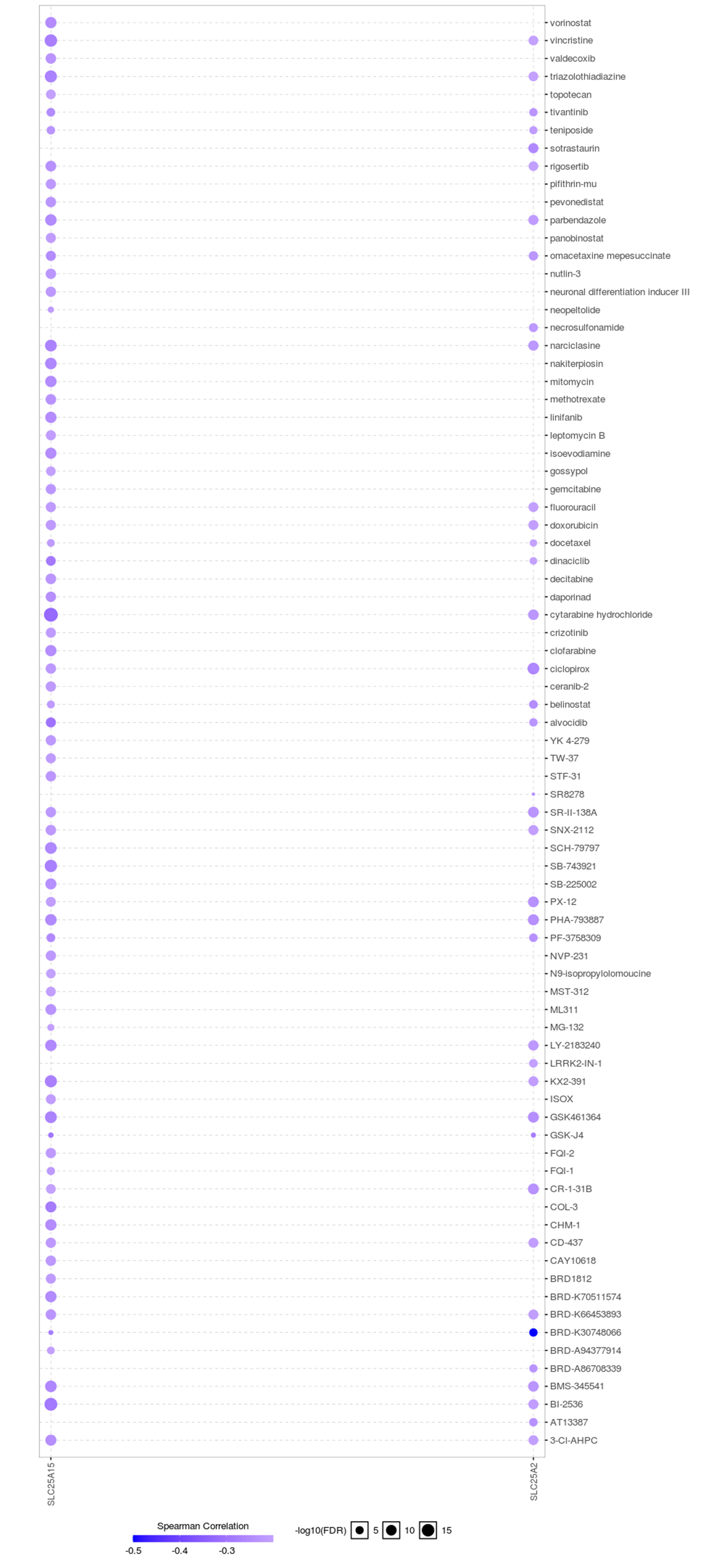

Supplement: Supplementary file 1 — Figure S1 [file CAM4-12-5035-s002.tif]

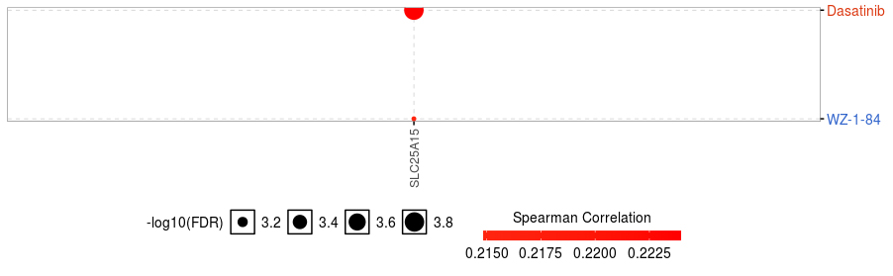

Supplement: Supplementary file 2 — Figure S2 [file CAM4-12-5035-s004.jpg]

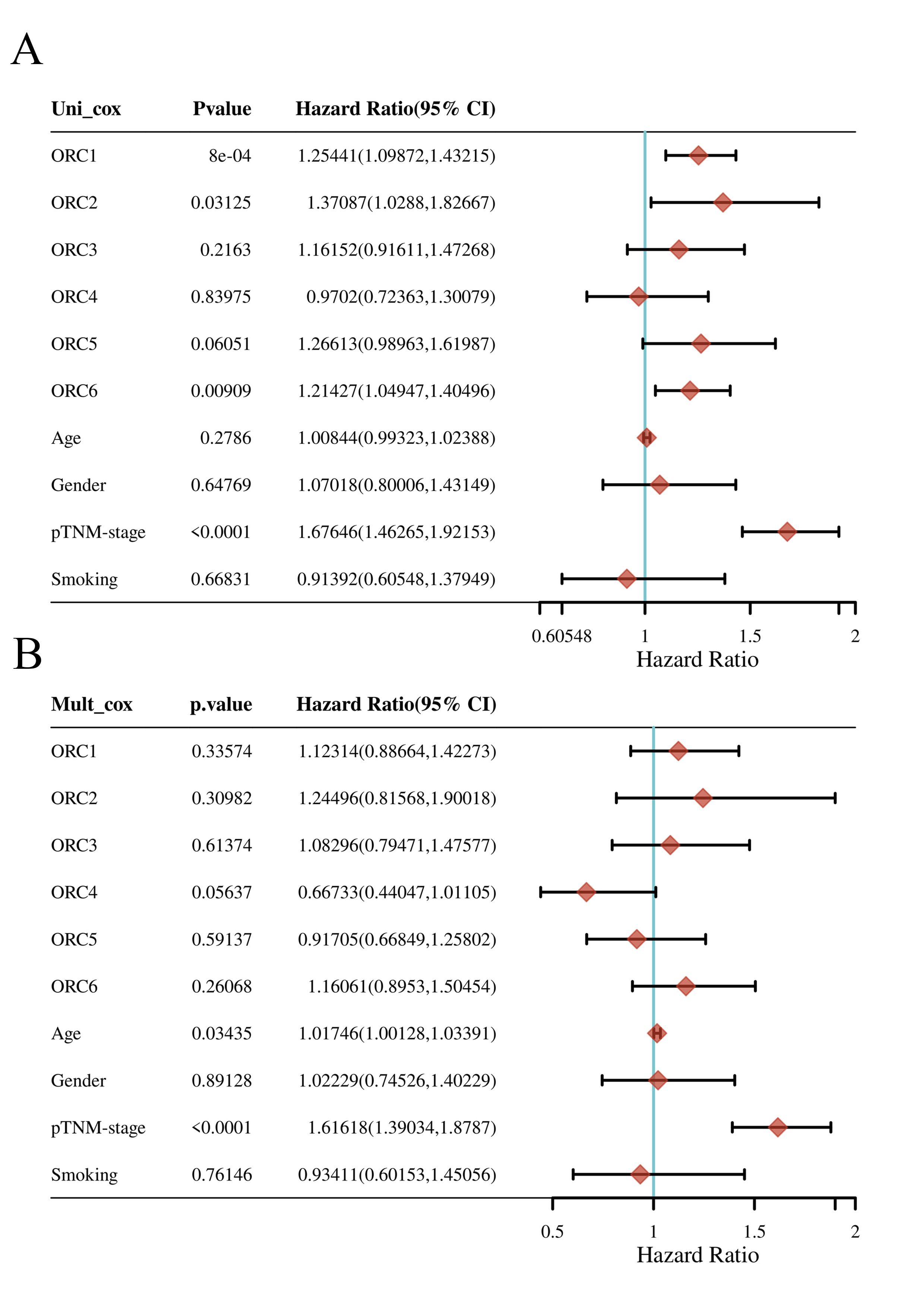

Supplement: Supplementary file 3 — Figure S3 [file CAM4-12-5035-s003.jpg]
